# Structome-TM: complementing dataset assembly for structural phylogenetics by addressing size-based biases

**DOI:** 10.1093/bioadv/vbag035

**Published:** 2026-02-17

**Authors:** Ashar J Malik, David B Ascher

**Affiliations:** School of Chemistry and Molecular Biosciences, The University of Queensland, Brisbane, QLD, 4072, Australia; Australian Centre for Ecogenomics, The University of Queensland, Brisbane, QLD, 4072, Australia; Computational Biology and Clinical Informatics, Baker Heart and Diabetes Institute, Melbourne, VIC, 3004, Australia; School of Chemistry and Molecular Biosciences, The University of Queensland, Brisbane, QLD, 4072, Australia; Australian Centre for Ecogenomics, The University of Queensland, Brisbane, QLD, 4072, Australia; Computational Biology and Clinical Informatics, Baker Heart and Diabetes Institute, Melbourne, VIC, 3004, Australia

## Abstract

**Motivation:**

Harnessing the explosion of protein structure data to uncover deep evolutionary relationships requires effective comparison methods. While widely used global alignment techniques are powerful, they can fail to identify homologous structures that differ significantly in size or domain architecture.

**Results:**

To address this limitation, we introduce Structome-TM, a web resource for assembling datasets for distance-based phylogenetic reconstruction. Making use of the Template Modelling score to prioritize local structural similarity, Structome-TM excels at identifying these otherwise obscured relationships, allowing users to build a comprehensive structural neighbourhood of proteins suitable for comparison. To facilitate this dataset assembly, the resource accepts Protein Data Bank identifiers, user-uploaded structure files, and protein sequences as inputs. When querying using a protein sequence, protein structures are predicted in real-time and their respective neighbourhoods determined, enabling analysis where experimentally determined structures may not be available. Through its user-friendly interface, Structome-TM provides a powerful and necessary approach for a more comprehensive exploration of protein evolution.

**Availability and implementation:**

This resource is freely available at: https://biosig.lab.uq.edu.au/structome_tm/.

## 1 Introduction

The large-scale availability of protein structure data has revolutionized the exploration of deep phylogenetic relationships (Malik *et al.* 2020, Puente-Lelievre *et al.* 2025). Because protein structure is conserved over much longer evolutionary distances than sequence (Illergård *et al.* 2009), it provides a powerful signal for detecting ancient homology that sequence-based methods alone can no longer identify (Malik 2018, Puente-Lelievre *et al.* 2025). These relationships are typically determined in a two-part process involving: (i) gathering structures to define the taxon set and (ii) comparing these structures to infer evolutionary relationships. The Structome suite is intended to streamline this process. The first tool developed in this suite, Structome-Q [originally published as Structome (Malik *et al.* 2023)], uses the Q-score metric from GESAMT (Krissinel 2012) to assess global structural similarity. It efficiently identifies related proteins and performs the necessary pairwise comparisons to construct a neighbour-joining tree.

The Q-score metric is defined as:


$$Q=\frac{N_{\text{align}}^{2}}{\left(1+{\left(\frac{\text{RMSD}}{R_{0}}\right)}^{2}\right)N_{1}N_{2}}$$

where $N_{\text{align}}$ is the number of aligned residue pairs, RMSD is the root-mean-square deviation of the alignment, $R_{0}$ is a scaling parameter (set to 3.0 Å), and $N_{2}$ are the residue counts of the two structures.

The normalization by the product of the protein lengths ($N_{1}N_{2}$) makes the Q-score a robust measure of global structural similarity. However, this inherently penalizes alignments where structures share only localized regions of similarity, such as proteins with partial domain matches. As a result, these biologically significant homologues may rank poorly and could be overlooked.

In contrast, the TM-score (Zhang and Skolnick 2005) adopts a different approach:


$$\text{TM-score}=\frac{1}{L_{\text{target}}}\sum_{i=1}^{N}\frac{1}{1+{\left(\frac{d_{i}}{d_{0}(L_{\text{target}})}\right)}^{2}}$$

where $L_{\text{target}}$ is the length of the target protein, *N* is the number of aligned residues, $d_{i}$ is the distance between aligned residue pairs, and


$$d_{0}(L_{\text{target}})=1.24\sqrt[{3}]{L_{\text{target}}-15}-1.8,$$

The implementation of this metric within Foldseek (van Kempen et al. 2022) prioritizes local similarity by normalizing for alignment length rather than overall protein size. This is particularly advantageous for identifying meaningful alignments between multi-domain proteins that may only share a single domain with the query, highlighting relationships underestimated by the Q-score. The latest resource in this suite, Structome-TM, therefore provides this powerful local alignment methodology. By offering complementary search strategies, the Structome suite provides researchers with a more comprehensive and nuanced toolkit for structural phylogenetics.

The primary utility of Structome-TM is as an end-to-end workflow for phylogenetic dataset assembly. A typical metric integrated in a web app can query structural databases and returns a ranked hits list, leaving users to curate taxa and build trees using external resources (Holm 2022, van Kempen *et al*. 2022). Structome-TM streamlines this process by (i) annotating hits with SCOP, CATH, ECOD, Pfam, and NCBI taxonomy, (ii) providing an interactive interface to select a custom taxon set, and (iii) generating and visualizing a neighbour-joining tree on-the-fly from symmetrized TM-score distances, with Newick export. This integrated ‘search → curate → build tree’ pipeline is the key feature that distinguishes Structome-TM as a phylogeny-ready tool.

To clarify the distinctions between Structome-Q and Structome-TM, their features are summarized in Table 1.

**Table 1:** Comparison of Structome-Q with Structome-TM.^a^

Feature	Structome (Original)	Structome-Q	Structome-TM (this work)
Primary Metric	Q-score	Q-score	**TM-score**
Core Engine	GESAMT	GESAMT	**Foldseek**
Alignment Focus	Global Similarity	Global Similarity	**Local Similarity**
Input Type	PDB ID	PDB ID	PDB ID/**Sequence/Structure**
Taxon set	Pre-computed (top 50)	User-defined	User-defined
Status	Superseded	Active	**New**

a The table highlights the key differences in methodology and functionality between the original published tool, the updated Structome-Q, and the new Structome-TM.

Bold values indicate features that are new or distinct to Structome-TM.

## 2 Methods

The architecture of Structome-TM builds upon the framework established by Structome-Q (Malik *et al.* 2023). The methodology can be divided into three main components: dataset construction, the search algorithm, and the web server implementation.

### 2.1 Dataset construction

The foundation of Structome-TM is a representative, non-redundant set of protein structures derived from the RCSB Protein Data Bank (Berman *et al.* 2002) (PDB; https://www.rcsb.org). To curate this dataset, all PDB entries were first filtered to remove short peptides, retaining only proteins longer than 50 amino acids. This collection was then clustered at 90% sequence identity using USEARCH (Edgar 2010). Each resulting cluster is represented by a single centroid structure, which serves as a proxy for all its members. This approach makes the subsequent all-versus-all comparison computationally tractable.

This process yields a final, non-redundant centroid dataset of 69 138 structures.

### 2.2 Structural search and analysis

To enable rapid querying, an all-versus-all pairwise comparison of all centroids in the database was performed using Foldseek (van Kempen *et al*. 2022), with the resulting TM-scores stored. When a user submits a PDB structure as a query, it is first mapped to its pre-calculated cluster centroid for which the pre-computed results are returned.

To broaden accessibility, Structome-TM also accepts a protein sequence or user-provided single-chain protein structure in either CIF or PDB formats. In case of a sequence, it is first folded into a 3D model in real-time using ESMFold (Lin *et al.* 2023). This prediction, which is limited to sequences up to 300 amino acids, requires ESMAtlas API functionality which may not always be guaranteed. The ‘Upload structure’ feature provides a drop-in fallback during any periods of downtime. The resulting predicted structure is then used as the query against the full RCSB PDB. In addition to returning hits, a dedicated column is populated informing if the target protein is a member of the core Structome-TM dataset. Additionally, results for both structure- and sequence-based query are annotated with data from several key resources, including UniProt (UniProt Consortium 2015), Pfam (Mistry *et al.* 2021), SCOP (Fox *et al.* 2014), CATH (Sillitoe *et al.* 2021), ECOD (Schaeffer *et al.* 2017), and the NCBI taxonomy database (Schoch *et al.* 2020).

### 2.3 Web server implementation

The web application is built using the Flask Python framework and is deployed within Docker containers managed by an Nginx web server. The front-end is developed with standard HTML, CSS, and JavaScript, utilizing AJAX. Interactive 3D visualization of protein structures is rendered using the Mol* viewer (Sehnal *et al.* 2021). For phylogenetic analysis, neighbour-joining trees are generated from the ‘1 − TM-score’ distance metric using Biopython and are rendered as interactive diagrams in the browser via the D3.js library. Because TM-score is directional, distances are symmetrized as $d=1-\frac{1}{2}(\text{TM}(\text{query}\to \text{target})+\text{TM}(\text{targe}\to \text{tquery}))$.

This transformation is a standard method to convert a similarity score into a dissimilarity (distance) metric suitable for clustering algorithms like neighbour-joining. The resulting topology therefore reflects phenotypic (structural) relationships. While this provides a powerful hypothesis of structural relationships, statistical support measures (e.g. bootstrapping) are not generated on-the-fly. Such analyses require computationally intensive pseudo-replicate sampling (e.g. via molecular dynamics simulations) that is infeasible for a real-time web app, and discussed later.

Performance varies by query type: pre-computed (PDB ID) searches return in under a few seconds. Live structure-based searches (Custom Upload) and sequence-based searches against the full PDB typically complete in 10 seconds and 20–30 seconds, respectively, with the latter gated by ESMAtlas API latency.

### 2.4 Benchmarking methodology

To quantitatively benchmark the performance of Structome-TM against Structome-Q, we generated a direct comparison dataset from their respective precomputed all-versus-all centroid results. First, for each query, we identified the intersection of hits found by both methods (Foldseek and GESAMT). This common subset of pairs, representing an ‘apple-to-apple’ comparison, was then annotated with SCOP superfamily classifications. To ensure a stringently labelled dataset, any pair where either the query or the target lacked a SCOP annotation was excluded from the analysis. The remaining pairs were then classified: ‘Positives’ (homologous) were defined as pairs where the query and hit share the same SCOP superfamily (e.g. ‘a.1.1’ and ‘a.1.1’), and ‘Negatives’ (non-homologous) as pairs where they belong to different superfamilies (e.g. ‘a.1.1’ and ‘a.2.1’). This process yielded the final benchmark labelled dataset, comprising both similar sized and size-divergent protein pairs which were analysed and their score distributions visualized. Furthermore, a curated subset of ‘Positive’ pairs is available for interactive inspection on the ‘Benchmark’ page of this web app. This subset was selected by filtering for high-disagreement pairs (TM-score ≥0.8 and Q-score ≤0.2) and was sampled to ensure a diverse representation of superfamilies.

## 3 Usage

Structome-TM offers three query modes, RCSB PDB structure-based, custom structure-based and sequence-based, each tailored for different input-types, but all leading to a common interface for interactive analysis and phylogenetic tree generation.

### 3.1 Structure-based query

When a PDB identifier is submitted, the search is performed against the curated set of Structome-TM centroids. Results are displayed in a sortable table listing hits with a TM-score >0.1. Clicking any row expands it to show the full list of PDB IDs within that cluster and displays an interactive 3D superposition of the query structure against the hit centroid using the Mol* visualizer.

### 3.2 Custom structure query

Users can upload a custom structure file in PDB or CIF format. The server validates that the file contains a single protein chain of more than 50 residues before initiating a search against the full RCSB PDB.

### 3.3 Sequence-based query

For protein sequence submissions, the resource first predicts the query’s structure and then searches it against the complete PDB dataset (as of June 2025). The resulting table includes an additional, sortable column with a binary marker. This marker indicates whether a given hit is also a representative centroid within the core Structome-TM dataset.

### 3.4 Statistics and analysis

For all query modes, an accompanying histogram summarizes the distribution of TM-scores, providing a rapid overview of result quality. Users can then select hits via checkboxes to assemble a custom dataset for phylogenetic analysis. A key difference between the two modes is the composition of this dataset: the structure-based search allows only curated centroids to be used for tree reconstruction, whereas the sequence-based and custom structure searches allow any target protein to be included, regardless of its centroid status.

Upon submission, a neighbour-joining tree is generated using ‘1 − TM-score’ as the distance metric. The final tree is rendered as an interactive diagram, allowing inspection of individual leaf labels and download in Newick format for further annotation.

## 4 Case study and user guidance

**Figure 1:**
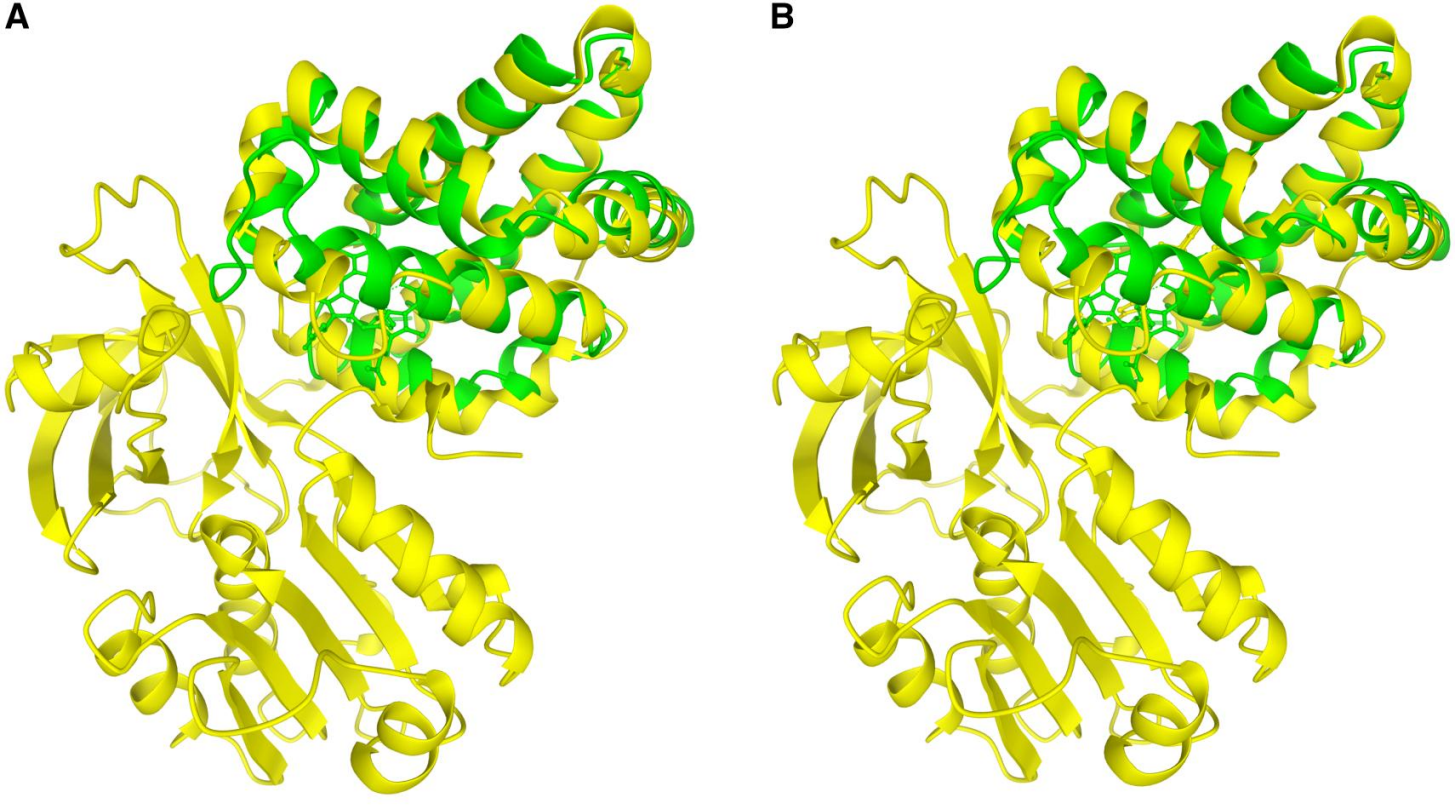
Protein structure alignment between haemoglobin from *Anser indicus* (RCSB PDB accession 1a4f, chain A, 141 amino acids, smaller structure) and flavohaemoglobin from *Saccharomyces cerevisiae* (RCSB PDB accession 4g1b, chain A, 399 amino acids, larger structure). Panel (A) illustrates the alignment from Structome-Q using the Q-score metric, while panel (B) shows the alignment from Structome-TM using TM-score. The Q-score for this alignment is 0.213, whereas the TM-score is 0.803. The lower similarity score from Q-score reflects its global normalization accounting for overall protein sizes. A score of 0.213 indicates that this hit will probably be lost in the background noise.

To demonstrate how Structome-TM captures meaningful alignments missed by global methods, haemoglobin from *Anser indicus* (a single globin domain) was compared with flavohaemoglobin from *Saccharomyces cerevisiae* (Fig. 1). Flavohaemoglobin is a larger, multi-domain protein, comprising a globin domain fused to an FAD-binding domain (Schuster *et al.* 2024).

Due to this size mismatch, the global alignment approach assigns a low Q-score of 0.213. This score is low enough to be indistinguishable from background noise, meaning this significant homologous relationship would likely be missed.

In contrast, by focusing on the shared local similarity, a high TM-score of 0.803 is obtained. This result demonstrates how Structome-TM can successfully build a more comprehensive structural neighbourhood. It ensures that valuable homologous relationships within multi-domain proteins are correctly identified, enabling more complete and accurate downstream phylogenetic analyses.

### 4.1 Quantitative benchmarking of score distributions

**Figure 2:**
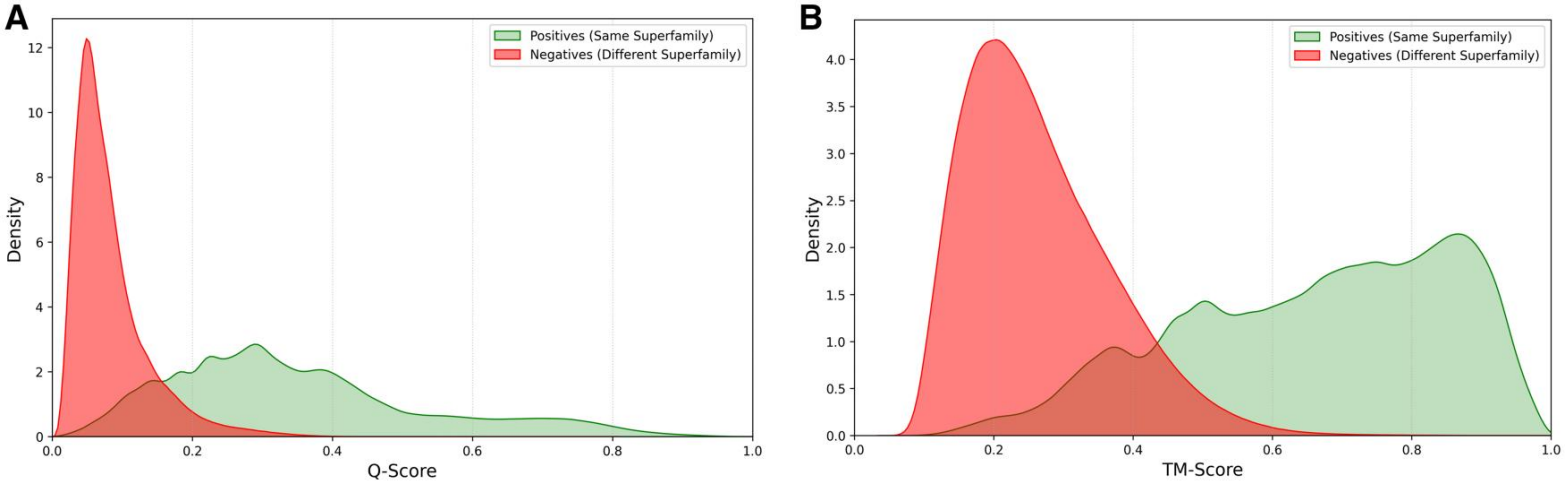
Density plots of Q-score (panel A) and TM-score (panel B) for positive (right; pairs sharing a SCOP superfamily) and negative (left; pairs from different SCOP superfamilies) hits from the intersection dataset. The Q-score signal is ‘degraded’ and shifted to the left due to size-based penalties, whereas the TM-score provides a much more practical, high-scoring signal for true positives.

The results of our benchmark (Fig. 2) reveals the practical difference between the two metrics. The score distributions show that TM-score provides a higher actionable signal.

The Q-score distribution for true positives (Fig. 2A) is ‘degraded’ by size-based biases; it is low, broad, and peaks at a score of ∼0.3. In contrast, the TM-score distribution for true positives (Fig. 2B) is pushed to the right, with a clear separation from the negative distribution and a strong peak at ∼0.9.

### 4.2 Practical implications for dataset assembly

The difference in score distributions seen in Fig. 2 has critical, practical implications for real-world dataset assembly. In a typical use case, a user must set a single score threshold to separate a meaningful signal from background noise.

The Q-score distribution (Fig. 2A) makes this task difficult. The signal from true positives (green) is ‘degraded’ by size-based penalties and peaks at a low score (≈0.3). The TM-score (Fig. 2B), in contrast, is robust to these penalties. It provides a high-scoring signal for true positives, creating a clear separation between the two distributions.

### 4.3 Interpreting structural hits

The Structome-TM results table is intentionally enriched with annotations from multiple databases. This design allows users to effectively interpret their search results. Classifications from SCOP, CATH, and ECOD, for example, provide additional evidence of shared structural relatedness.

In contrast, the Pfam annotation classifies proteins by sequence similarity. A key workflow in Structome-TM is to compare these different annotations. The appearance of multiple Pfam IDs for hits that share the same structural classification (e.g. SCOP Superfamily) is a strong proxy for deep evolutionary relationships. It highlights proteins that are structurally similar but have diverged at the sequence level to the point where sequence-based methods would fail to group them.

We can demonstrate this workflow with a haemoglobin query (PDB accession 1a4f, chain A), which belongs to the Globin Pfam family (Pfam accession PF00042). As expected, hits in the highest-scoring bins (TM-score ≥0.8) are almost exclusively other globins sharing the same Pfam accession PF00042.

In the 0.6≤TM-score<0.7 range, new Pfam accessions appear, such as Protoglobin (PF11563) and the N-terminal domain of the RsbR stressosome regulator (Pfam accession PF08678). This latter hit is a prime example of the workflow’s utility: despite its completely different sequence family and function, the structural link is a verified deep homologous relationship where the N-terminal domain of RsbR is a ‘recycled’ globin fold (Murray *et al.* 2005).

*Disclaimer:* This workflow must be guided by supporting evidence, as high structural similarity does not always equal homology (shared ancestry). Structural analogs may produce high similarity scores without necessarily sharing a common ancestor. We note that many of these deep homologous relationships, including the one between Globin (PF00042) and RsbR (PF08678), are also captured by the Pfam database’s ‘Clan’ groupings. Pfam clans are curated groupings that often use additional evidence, such as profile HMMs and known structural relatedness, to connect families that conventional sequence similarity alone cannot link (Finn *et al.* 2006). Our choice of a query from a well-studied protein family is to demonstrate how direct querying of Structome-TM, can computationally rediscover these established, complex relationships from a single entry, providing a complementary approach to curated database exploration. The purpose of Structome-TM remains to streamline dataset assembly by providing a strong structural hypothesis. The user’s role is to then use the provided annotations and supporting literature to curate a final, phylogenetically meaningful dataset.

#### 4.3.1 Topology reliability and support

The ‘1−TM’ transformation addresses scaling (similarity → dissimilarity) but does not by itself provide statistical support. When support values are desired, users can generate replicate distance matrices by resampling the structural signal and re-inferring trees, as in previous works (Malik 2018, Malik *et al.* 2020). In practice, this can be done by creating small structural ensembles per protein (e.g. Molecular Dynamics/Monte Carlo simulation based perturbations, and now increasingly with generative models) and recomputing TM-scores between randomly selected structural representatives per replicate. Replicate trees, then, yield clade frequencies that function as bootstrap-like supports.

## 5 Outlook

Structome-Q and Structome-TM are designed to streamline the initial, and often most challenging, step of structural phylogenetics: the assembly of a relevant protein structure dataset. This dataset assembly is the essential first step for using both distance-based methods included within these resources and character-based phylogenetic methods, which use 3Di structural characters to infer maximum likelihood trees (Puente-Lelievre *et al*. 2023) and helper tools like Structome-AlignViewer (Malik *et al*. 2026). Thus, while these resources provide a direct route to distance-based trees, their fundamental purpose is to empower researchers with the curated data needed for any downstream structural evolutionary analysis.

Looking ahead, the data exploration process will be enhanced by incorporating advances in generative models. Future developments will explore the use of protein language model (pLM) embeddings as an alternative feature for structural comparison, alongside the planned integration of generative AI. This latter capability will enable automated textual summaries of search results, immediately highlighting common functional or taxonomic features within a structural neighbourhood. Such enhancements promise to transform this resource into an interactive knowledge discovery platform, accelerating insights into the evolution of protein structure and function.

## Data Availability

Structome-TM is available at https://biosig.lab.uq.edu.au/structome_tm/.
